# Mesial temporal tau pathology impacts basal forebrain degeneration in early Alzheimer's disease

**DOI:** 10.1002/alz.71050

**Published:** 2025-12-26

**Authors:** Ying Xia, Matthew W. Dean, Pierrick Bourgeat, Natasha Krishnadas, Paul Maruff, Colin L. Masters, Victor L. Villemagne, Jurgen Fripp, Christopher C. Rowe, Vincent Doré, Elizabeth J. Coulson

**Affiliations:** ^1^ The Australian e‐Health Research Centre CSIRO Health and Biosecurity Herston Queensland Australia; ^2^ School of Biomedical Sciences The University of Queensland, Brisbane St. Lucia Queensland Australia; ^3^ Department of Molecular Imaging & Therapy Austin Health Heidelberg Victoria Australia; ^4^ The Florey Institute of Neuroscience and Mental Health The University of Melbourne Parkville Victoria Australia; ^5^ Cogstate Ltd. Melbourne Victoria Australia; ^6^ Department of Psychiatry University of Pittsburgh Pittsburgh Pennsylvania USA; ^7^ The Australian e‐Health Research Centre CSIRO Health and Biosecurity Parkville Victoria Australia; ^8^ Queensland Brain Institute The University of Queensland St. Lucia Queensland Australia

**Keywords:** [^18^F]MK‐6240, Alzheimer's disease, amyloid imaging, basal forebrain volume, hippocampal volume, magnetic resonance imaging, positron emission tomography, tau pathology

## Abstract

**INTRODUCTION:**

The cholinergic basal forebrain system, particularly the nucleus basalis of Meynert (Ch4), is selectively vulnerable to amyloid beta (Aβ) and tau in Alzheimer's disease (AD). Their interplay may be a critical driver of AD progression, but remains poorly understood.

**METHODS:**

Data from 779 older individuals in the Australian Imaging, Biomarker, and Lifestyle study were analyzed, all of whom underwent ^18^F‐NAV4694 Aβ and ^18^F‐MK6240 tau positron emission tomography and magnetic resonance imaging.

**RESULTS:**

The co‐occurrence of Aβ and mesial‐temporal (MTL) tau pathologies was associated with reduced Ch4 volumes in cognitively unimpaired individuals. MTL tau burden was associated with Ch4 volumes exclusively in cognitively unimpaired individuals with established Aβ pathology, which was not observed for the hippocampus. This association persists in individuals with mild cognitive impairment, but was not apparent in AD dementia.

**DISCUSSION:**

Findings underscore early Ch4 degeneration associated with Aβ and tau pathologies, supporting potential cognitive benefits of cholinergic therapies in early disease stages.

**HIGHLIGHTS:**

- Early‐stage tau pathology in the mesial‐temporal (MTL) region was assessed using ^18^F‐MK6240 positron emission tomography.
- Co‐occurring amyloid beta and MTL tau was linked to reduced nucleus basalis of Meynert (Ch4) volume in cognitively unimpaired individuals.
- Ch4 volume was associated with MTL tau burden exclusively in preclinical Alzheimer's disease (AD).
- No comparable association was observed between MTL tau and hippocampal volume.
- The MTL tau–Ch4 association persisted into the prodromal stage of AD.

## 1 BACKGROUND

Alzheimer's disease (AD) is a progressive neurodegenerative disorder characterized by early accumulation of amyloid beta (Aβ) plaques, decades before dementia onset,^1^, ^2^ and the subsequent spread of tau neurofibrillary tangles (NFTs), which correlates closely with neurodegeneration and cognitive decline.^3^, ^4^ Histological studies indicate that the formation of NFTs in AD follows a relatively stereotyped regional progression, beginning in the trans‐entorhinal and entorhinal regions before spreading neocortically, typically in the presence of established cortical Aβ deposition.^5^, ^6^ Emerging evidence suggests that early tau accumulation may occur in subcortical regions, such as the locus coeruleus and nucleus basalis of Meynert (referred to as the Ch4 subregion of the basal forebrain [BF]).^7^, ^8^ The Ch4 is the major source of cortical cholinergic innervation and highly vulnerable to Aβ and tau pathologies, leading to early cholinergic disruptions that precede and accelerate cognitive decline.^9^, ^10^ Together, Aβ accumulation, tau pathology, and BF cholinergic degeneration engage in a three‐way interplay that may be a critical driver of disease progression in AD.^11^ However, this tripartite interaction remains poorly understood, with the role of tau pathology in BF degeneration particularly underexplored.

In vivo neuroimaging studies have measured atrophy in the BF as a surrogate marker of cholinergic degeneration, which is associated with AD pathology.^12^, ^13^ These studies show that BF atrophy is associated with Aβ burden in preclinical and clinical stages of AD,^14^, ^15^, ^16^ with Aβ‐related volume loss being greater in the Ch4^15^, ^17^ and occurring prior to degeneration in the entorhinal cortex.^18^, ^19^ Early Aβ‐related BF atrophy is also associated with decline in memory and attention, beyond that explained by hippocampal atrophy.^20^ However, Aβ is not the sole contributor to BF degeneration; *post mortem* studies show that Ch4 cholinergic neurons exhibit selective vulnerability to tau pathology,^8^ with this relationship confirmed in humans where Ch4 volume loss has been identified in cognitively unimpaired (CU) individuals with elevated cerebrospinal fluid levels of phosphorylated tau.^21^ Additionally, tau biomarkers assessed by plasma assays and positron emission tomography (PET) predict the rate of BF atrophy.^22^, ^23^ Few studies have examined the effects of both Aβ and tau pathologies on BF cholinergic degeneration. While these pathologies interact and co‐evolve during AD progression, they may affect BF atrophy through distinct mechanisms,^11^ and disentangling their individual contributions remains highly challenging.

Examination of Aβ, early‐stage tau pathology, particularly within the mesial temporal lobe (MTL), and BF volume in CU older individuals could provide greater understanding of the selective vulnerability of the BF in AD. Notably, Aβ and tau pathologies exhibit less covariance in the preclinical stage than in later clinical stages, reflecting the progressive nature of AD.^3^ The PET tracer ^18^F‐MK6240 shows a better ability to detect early tau accumulation as NFTs in CU individuals,^24^ typically exhibiting higher PET retention in the MTL among CU individuals with abnormal Aβ levels (Aβ+), before progressing to the temporo‐parietal (TE) region.^25^ This capacity enables precise mapping of tau pathology and facilitates investigations into its relationships with BF degeneration and Aβ pathology. Given the strong association between tau pathology and neurodegeneration, hippocampal volume is also considered a key structural marker, as it closely correlates with tau pathology and memory loss.^4^, ^26^

In this study, we investigate the relationships among Aβ burden, tau pathology, and volumes of Ch4 and hippocampus in the Australian Imaging, Biomarker and Lifestyle (AIBL) study cohort. Given the well‐established link between Aβ and Ch4 in preclinical AD,^14^, ^15^, ^16^ we first examined whether this relationship varies based on tau status in CU older individuals. We then evaluated the direct associations between tau burden, with emphasis on the MTL, and Ch4 and hippocampal volumes across different AD stages.

## 2 METHODS

### 2.1 Participants

**RESEARCH in CONTEXT**

- **Systematic Review**: The authors conducted a PubMed search to identify in vivo imaging studies examining the relationships between amyloid beta (Aβ), tau pathology, and basal forebrain volume in Alzheimer's disease (AD). However, the complex interplay among these processes remains poorly understood.
- **Interpretation**: Our findings show that the co‐occurrence of Aβ and mesial‐temporal (MTL) tau was associated with early degeneration of the nucleus basalis of Meynert (Ch4) in cognitively unimpaired (CU) individuals. Early MTL tau pathology, alongside Aβ, plays an important role in Ch4 degeneration during the preclinical and prodromal stages of AD. However, in the absence of established Aβ pathology, MTL tau was not linked to Ch4 degeneration in aging CU individuals.
- **Future Directions**: Future research should validate these findings in larger, more diverse cohorts, and further elucidate the onset and progression of basal forebrain cholinergic degeneration in association with AD progression and cognitive decline.

Participants from the AIBL study who underwent PET imaging for brain Aβ and tau assessments, as well as magnetic resonance imaging (MRI), were included. Most participants (≈ 93.7%) completed all three imaging assessments within a 6 month period.

AIBL is a large prospective cohort study of aging, in which participants > 60 years old are recruited from two study centers in Australia and undergo comprehensive neuroimaging, biomarker, and neuropsychological assessments at 18 month intervals.^27^, ^28^ Exclusion criteria include a history of non‐AD dementia, Parkinson's disease, schizophrenia, bipolar disorder, obstructive sleep apnea, serious head injury, current depression, or a high cardiovascular disease burden (including symptomatic stroke, uncontrolled diabetes, or current regular alcohol use above two standard drinks per day for women or four per day for men). Ethics approval for the AIBL study was obtained from the institutional ethics committees of Austin Health, St. Vincent's Health, Hollywood Private Hospital, and Edith Cowan University. Written informed consent was obtained from all participants before participation and at each visit.

### 2.2 Brain imaging

Aβ PET scans were acquired using the radiotracer ^18^F‐NAV4694, with a 20 minute scan performed at 50 minutes post‐bolus injection of 200 MBq (± 10%). Tau PET scans were acquired using the radiotracer ^18^F‐MK6240, with a 20 minute scan performed at 90 minutes post‐bolus injection of 185 MBq (± 10%).

High‐resolution structural MRI scans were acquired using a T1‐weighted anatomical magnetization‐prepared rapid gradient echo (MPRAGE) sequence, with the majority (88.7%) using acquisition parameters: repetition time = 2300 ms, echo time = 2.91 or 2.98 ms, flip angle = 9°, and voxel size = 1×1×1 mm^3^.

### 2.3 Image data processing

Aβ ^18^F‐NAV4694 PET scans were automatically analyzed without spatial smoothing using the MRI‐less Computational Analysis of PET from AIBL (CapAIBL),^29^ and estimated in Centiloid (CL) values using the non‐negative matrix factorization approach.^30^ Abnormal levels of Aβ burden (Aβ+) were defined using a threshold of 20 CL, as validated using autopsy data.^31^

Tau ^18^F‐MK6240 PET scans, without spatial smoothing, were spatially normalized to a standard template using CapAIBL, and the cerebellum cortex was used as the reference region to calculate the standardized uptake value ratio (SUVR). Tracer retention was measured in two regions of interest: the MTL region, comprising the entorhinal cortex, amygdala, hippocampus, and parahippocampus, and the TE region, comprising inferior and middle temporal, fusiform, supramarginal and angular gyri, posterior cingulate/precuneus, superior and inferior parietal, and lateral occipital cortex.^32^ Tau SUVR values in the MTL and TE were further expressed in the CenTauR unit, a universal standard scale.^32^ Abnormal CenTauR values were defined as values ≥ 1.5 standard deviations (SD) above the mean for the reference group of CU Aβ− individuals in the MTL (MTL+) and ≥ 2.0 SD in the TE (TE+).^33^, ^34^

Volumetric measures of the BF and hippocampus were quantified from MRI using a Statistical Parametric Mapping–based processing pipeline.^15^ MRI scans were analyzed for brain tissue segmentation and spatial normalization using computational anatomy toolbox (CAT12).^35^ The hippocampal volume was quantified across the left and right hippocampal regions, as identified using the Neuromorphometrics atlas. The gray matter segmentation was normalized to a pre‐generated population template and modulated to correct for volumetric changes introduced by spatial transformation, followed by a 4‐mm Gaussian smoothing. The BF subregions were identified in the template space using a stereotactic mask of the bilateral BF,^12^ allowing for volumetric assessment of the Ch4 subregion and its posterior subdivision (Ch4p).

Multi‐scanner effects on volumetric measures were corrected using ComBat,^36^ followed by adjustment for total intracranial volume (TIV), age, and sex using regression coefficients estimated from the reference group of CU Aβ− MTL− individuals. This pre‐adjustment of volumetric measures prior to statistical analysis helps minimize potential confounding effects of demographic variables (age, sex) on associations between tau burden and brain volumes.

### 2.4 Statistical analysis

Participants were grouped based on their clinical disease severity using the Clinical Dementia Rating (CDR) global score: CDR = 0 for CU, CDR = 0.5 for mild cognitive impairment (MCI), and CDR ≥ 1 for AD. Group‐wise differences were assessed with one‐way analysis of variance tests for continuous data and *χ* ^2^ testing for categorized data. All analyses were conducted in the R environment (version 4.2.0). A *P* value of < 0.05 (two sided) was considered statistically significant.

To understand the complex relationships among Aβ, tau pathology, and Ch4 and hippocampal volumes, a series of correlation analyses were conducted first in CU older individuals, for whom Aβ and tau burden in the MTL exhibited less covariance than in later clinical stages. Spearman rank correlation coefficient ρ was used for assessing the correlations, as the data were not always normally distributed. With the pre‐adjustment of Ch4 and hippocampal volumes for TIV, age, and sex, no further covariates were included. While multiple correlation analyses were performed, multiple testing correction was not applied, as these analyses were hypothesis driven rather than an exhaustive, data‐driven exploration.

The associations between Aβ CL values and brain volumes were first examined in all CU individuals, as well as in subgroups stratified by MTL or TE tau status. This approach enables evaluation of how the associations between Aβ CL values and volumes of Ch4 and hippocampus vary at different stages of tau pathology, as indicated by the presence of abnormal CenTauR measures in the MTL and TE. It provides a more targeted alternative to standard regression models that include tau burden as a covariate.

Similarly, the associations between MTL CenTauR values and brain volumes were assessed in all CU individuals, as well as in subgroups stratified by Aβ status. This approach enables clearer interpretation of tau‐related effects on volumes of the Ch4 and hippocampus by minimizing confounding from concurrent Aβ pathology. TE tau burden was not considered, given tau pathology is generally confined to the MTL in preclinical stages. When a significant association was noted in individuals likely to have concurrent Aβ and tau pathology, simple mediation analysis was performed using the bmemLavaan R package^37^ to assess whether and to what extent the covariance of PET‐derived measures for both pathologies accounted for the correlation.

The associations between MTL CenTauR values and Ch4 and hippocampal volumes were further assessed in individuals with MCI and AD, stratified by Aβ status. This was also followed by mediation analysis to evaluate to what extent the covariance of Aβ and tau PET quantifications accounted for the observed correlations. To assess between‐group differences across the AD continuum, moderation analyses were also conducted with group as the moderator of the association between MTL CenTauR values and Ch4 or hippocampal volumes. Given that tau pathology may spread more extensively beyond the MTL in individuals with cognitive symptoms, these analyses were repeated using TE CenTauR measures to assess the relationships between later‐stage tau pathology and Ch4 and hippocampal volumes. Correlation analyses were also repeated using Ch4p volumes to examine subregional specificity within the basal forebrain.

Exploratory voxel‐wise analyses were conducted within the CU Aβ+, MCI Aβ+, and AD groups to further examine the spatial distribution of associations between regional tau burden and Ch4 and hippocampal volumes across different AD stages. Analyses were performed with tau PET SUVR as the dependent variable and Ch4 or hippocampal volume as the independent variable, and no additional variable was considered. Statistical significance was assessed using a voxel‐wise false discovery rate (FDR) correction at *P* < 0.05.

## 3 RESULTS

### 3.1 Cohort characteristics

A total of 779 participants from the AIBL study were included (mean age = 73.4 ± 7.0 years; 50.3% female; 49.3% carrying one or two copies of the apolipoprotein E [*APOE*] ε4 allele). Based on their CDR global scores, 335 were classified as CU, 383 as having MCI, and 61 as having AD. The AD group was confirmed to be Aβ+ based on their Aβ PET quantification. Table 1 summarizes the characteristics of different clinical disease severity groups.

**TABLE 1:** Participant characteristics.

	CU	MCI	AD
**No. of participants**	335	383	61
**Age, years, mean (SD)**	74.1 (6.3)	**73.0 (7.4)**	**72.2 (7.6)**
**Female, *n* (%)**	190 (56.7)	**173 (45.2)**	29 (47.5)
**Education, years, mean (SD)**	13.8 (2.9)	**12.7 (3.2)**	**11.8 (3.0)**
***APOE* ε4 carriage, *n* (%)**	130 (40.4)	**190 (53.5)**	**43 (71.7)**
**History of hypertension, *n* (%)**	114 (34.0)	**169 (44.1)**	**29 (47.5)**
**History of diabetes, *n* (%)**	37 (11.0)	**84 (21.9)**	**13 (21.3)**
**Body mass index, mean (SD)**	26.8 (4.5)	26.1 (4.3)	26.3 (5.3)
**MMSE, mean (SD)**	28.7 (1.2)	**25.7 (2.8)**	**20.7 (4.6)**
**Aβ burden, Centiloid, mean (SD)**	22.1 (42.0)	**65.6 (55.9)**	**103.0 (37.7)**
**No. of Aβ+, *n* (%)**	115 (34.3)	**259 (67.6)**	**61 (100.0)**
**Tau in MTL, CenTauR, mean (SD)**	8.8 (15.4)	**45.2 (43.8)**	**78.0 (44.8)**
**No. of MTL+, *n* (%)**	93 (27.8)	**260 (67.9)**	**54 (88.5)**
**Tau in TE, CenTauR, mean (SD)**	6.3 (9.5)	**34.5 (46.7)**	**83.2 (63.2)**
**Ch4 volume** ^a^ **, mL, mean (SD)**	0.237 (0.016)	**0.216 (0.024)**	**0.197 (0.021)**
**Ch4p volume** ^a^ **, mL, mean (SD)**	0.097 (0.007)	**0.086 (0.013)**	**0.075 (0.012)**
**Hippocampus** ^a^ **, mL, mean (SD)**	6.05 (0.37)	**5.41 (0.69)**	**4.75 (0.63)**

*Note*: Compared to the CU group, variables showing significant differences in the MCI or AD groups are highlighted in bold.

^a^ Ch4 and hippocampal volumes were pre‐adjusted for total intracranial volume, age, and sex based on reference samples of CU Aβ– MTL– individuals.

Abbreviations: Aβ, amyloid beta; Aβ+, abnormal amyloid beta burden (≥ 20 Centiloids); AD, Alzheimer's disease; *APOE*, apolipoprotein E; Ch4, nucleus basalis of Meynert; Ch4p, posterior subdivision of nucleus basalis of Meynert; CU, cognitively unimpaired; MCI, mild cognitive impairment; MMSE, Mini‐Mental State Examination; MTL, mesial temporal region; MTL+, abnormal mesial temporal region CenTauR measure; TE, temporo‐parietal region; SD, standard deviation.

### 3.2 Tau mediates the relationship between Aβ burden and Ch4 volume in CU individuals

**FIGURE 1:**
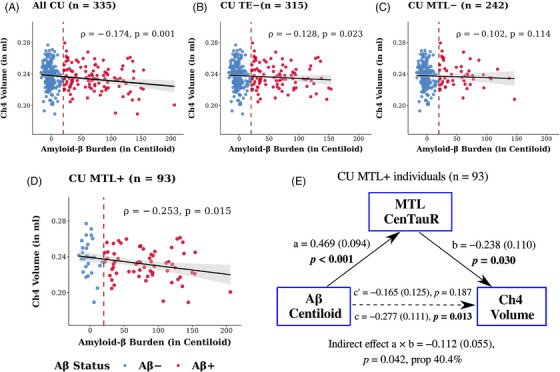
Associations between Aβ burden (in Centiloid) and Ch4 volume in (A) CU older individuals, (B) CU TE− individuals, and (C, D) subgroups stratified by MTL tau status. The red dashed lines illustrate the cut‐off value of 20 Centiloids applied for Aβ+. E, Mediation analysis examines if the observed association is explained by the covariance of Aβ Centiloid and MTL CenTauR values. The magnitude and direction of associations are represented by Spearman correlation coefficient *ρ*. The values *a*, *b*, and *c’* represent the standardized coefficients (standard errors) for specific pathways in the mediation model. *c* represents the total effect. Prop (in %) describes the proportion of the indirect effect relative to the total effect. Aβ, amyloid beta; Aβ+, abnormal amyloid beta burden; Ch4, nucleus basalis of Meynert; CU, cognitively unimpaired; MTL, mesial temporal region; MTL−, mesial temporal region CenTauR within normal limits; MTL+, abnormal mesial temporal region CenTauR measure; TE, temporo‐parietal region; TE−, temporo‐parietal region CenTauR within normal limits.

In CU individuals, regardless of tau status, Aβ CL values were negatively associated with Ch4 volumes (*ρ* = −0.174, *P* = 0.001; Figure 1A). This association remained statistically significant after exclusion of individuals with elevated tau PET retention in the typically later‐affected TE region (TE+; *n* = 315, *ρ* = −0.128, *P* = 0.023; Figure 1B).

When stratifying CU individuals by MTL tau status, the association between Aβ and Ch4 volumes was attenuated and not statistically significant in those with MTL CenTauR within normal limits (MTL−; *n* = 242, *ρ* = −0.102, *P* = 0.114; Figure 1C). In contrast, CU MTL+ individuals (*n* = 93), irrespective of tau status in the later‐affected TE region, showed a significant negative association (*ρ* = −0.253, *P* = 0.015; Figure 1D). Among those MTL+ individuals but with no TE involvement (TE−; *n* = 74), this association was attenuated and not statistically significant (*ρ* = −0.188, *P* = 0.108), likely due to limited statistical power despite a moderate effect size. No significant associations were found between Aβ and hippocampal volumes in CU individuals or subgroups stratified by tau status (Figure S1 in supporting information).

Given the prevalent co‐occurrence of Aβ and MTL tau pathology in CU MTL+ individuals, we tested whether the covariance of their PET quantifications contributed to the observed Aβ–Ch4 association. Mediation analysis showed that this association was largely explained by the covariance of Aβ CL and MTL CenTauR values, as indicated by a significant indirect effect (β[standard error (SE)] = −0.112[0.055], *P* = 0.042; Figure 1E), accounting for 40.4% of the total effect.

### 3.3 Association of MTL tau burden and Ch4 volume in CU individuals

**FIGURE 2:**
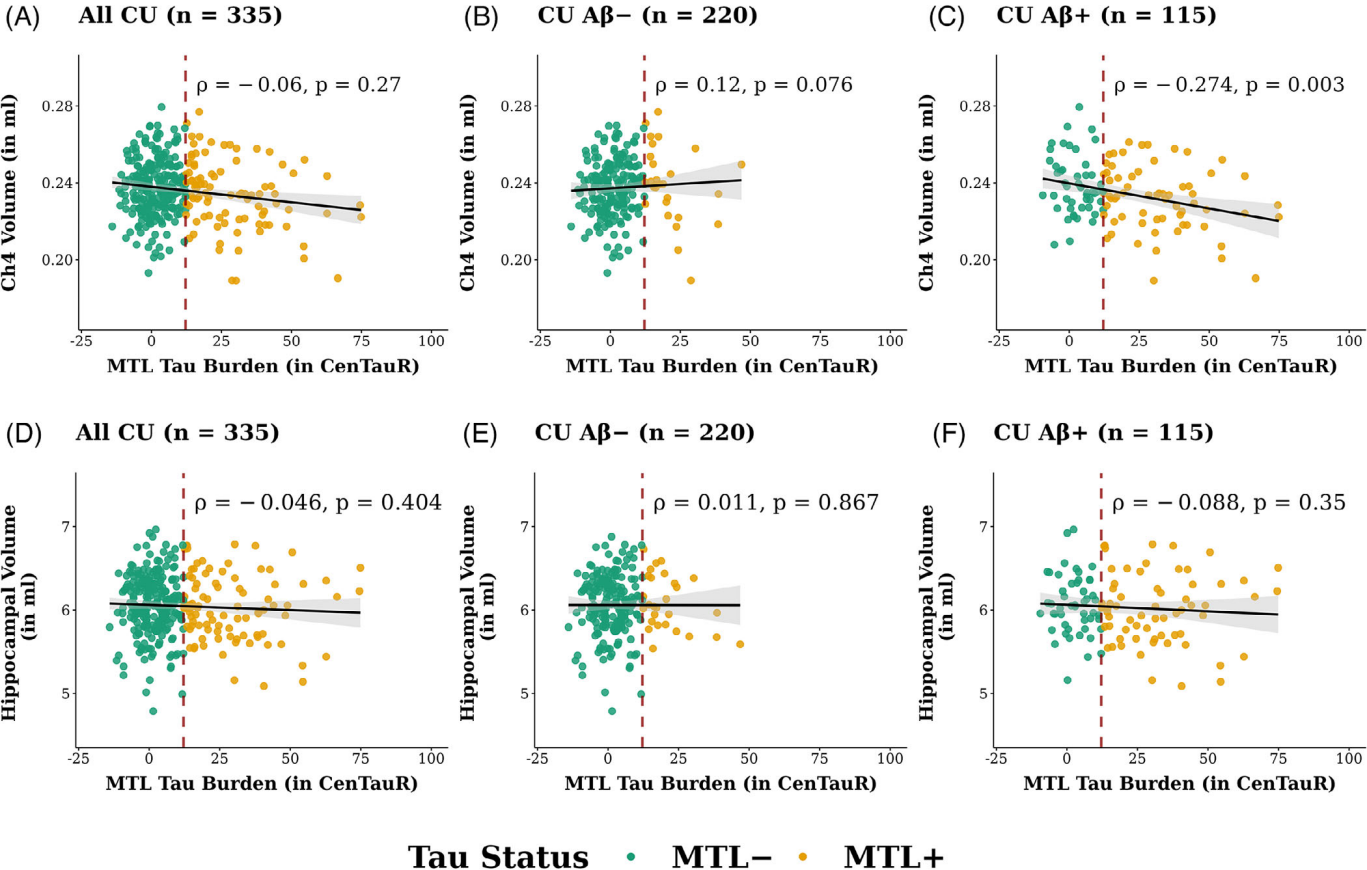
Associations between MTL CenTauR measures and (A−C) Ch4 volumes and (D−F) hippocampal volumes in CU older individuals and in subgroups stratified by Aβ status. The red dashed lines illustrate the cut‐off value for MTL+, estimated as 1.5 standard deviations above the mean of CU Aβ− individuals. The magnitude and direction of associations are represented by Spearman's correlation coefficient *ρ*. Aβ, amyloid beta; Aβ−, amyloid beta burden within normal limits; Aβ+, abnormal amyloid beta burden; Ch4, nucleus basalis of Meynert; CU, cognitively unimpaired; MTL, mesial temporal region; MTL−, mesial temporal region CenTauR within normal limits; MTL+, abnormal mesial temporal region CenTauR measure; TE, temporo‐parietal region; TE−, temporo‐parietal region CenTauR within normal limits.

Across all CU individuals, in whom tau pathology is generally confined to the MTL with limited involvement of the TE,^34^ no significant association was found between MTL CenTauR values and Ch4 volumes (*ρ* = −0.060, *P* = 0.270; Figure 2A). When individuals were stratified by Aβ status, Ch4 volumes were negatively correlated with MTL CenTauR values in CU Aβ+ individuals (*n* = 115, *ρ* = −0.274, *P* = 0.003; Figure 2C). In CU Aβ− individuals (*n* = 220), no significant association between MTL tau and Ch4 volumes was noted (Figure 2B). Again, no associations were observed between MTL CenTauR values and hippocampal volumes, in either all CU individuals or subgroups stratified by Aβ status (Figure 2D–F).

**FIGURE 3:**
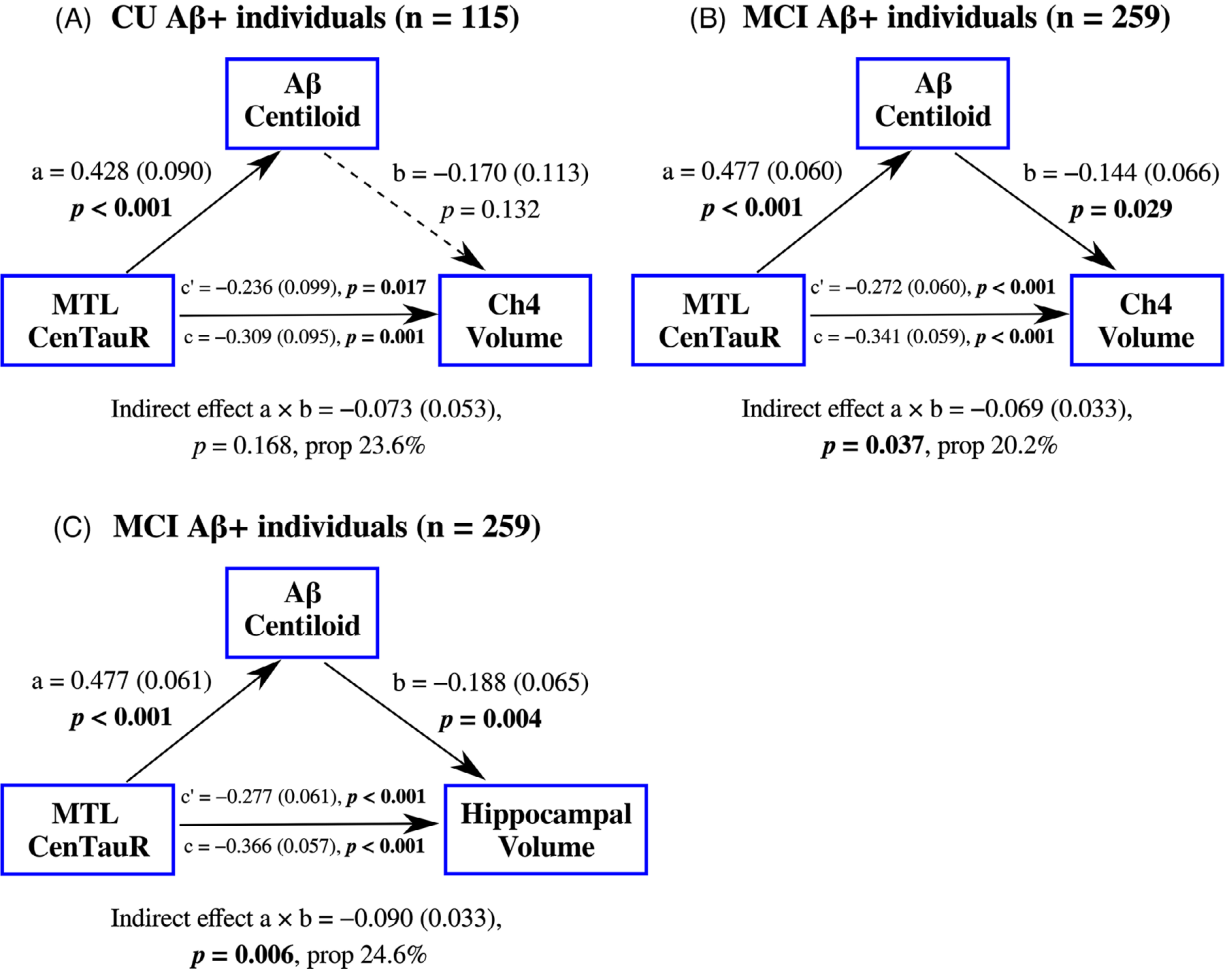
Mediation analysis examining if the observed associations between MTL CenTauR measures and volumes of Ch4 and hippocampus in (A) the CU Aβ+ and (B), (C) MCI Aβ+ groups are explained by the covariance of Aβ Centiloid and MTL CenTauR values. The values *a*, *b*, and *c’* represent the standardized coefficients (standard errors) for specific pathways in the mediation model. *c* represents the total effect. Prop (in %) describes the proportion of the indirect effect relative to the total effect. Aβ, amyloid beta; Aβ+, abnormal amyloid beta burden; Ch4, nucleus basalis of Meynert; CU, cognitively unimpaired; MCI, mild cognitive impairment; MTL, mesial temporal region

To assess whether the covariance of Aβ and tau PET quantifications contributed to the observed MTL tau–Ch4 association in CU Aβ+ individuals, mediation analysis indicated a non‐significant indirect effect through Aβ CL values (β[SE] = −0.073[0.053], *P* = 0.168), but revealed a significant direct effect of MTL CenTauR values on Ch4 volume (β[SE] = −0.236[0.099], *P* = 0.017; Figure 3A), accounting for 76.4% of the total effect.

### 3.4 Association of MTL tau burden and brain volume in symptomatic AD

**FIGURE 4:**
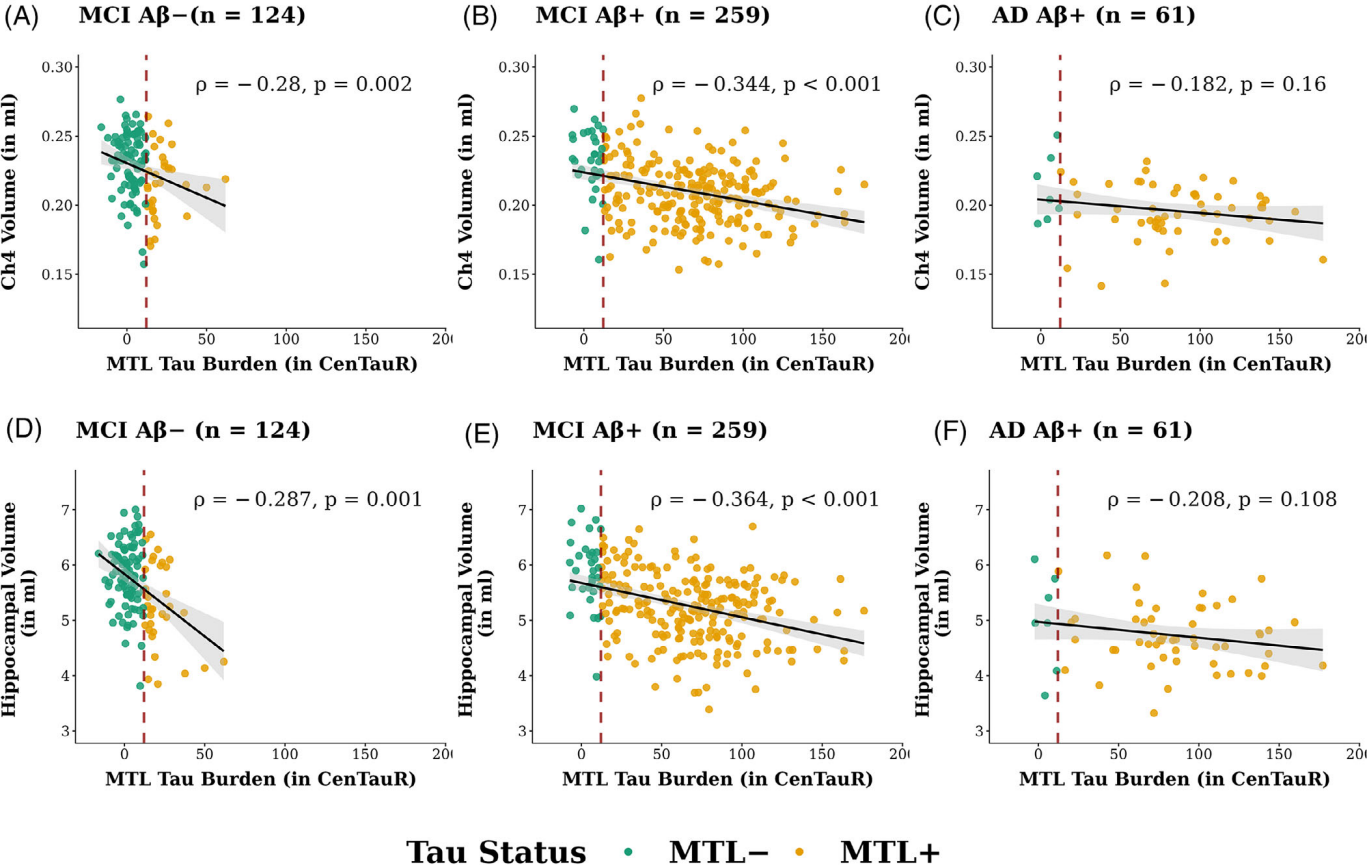
Associations between MTL CenTauR measures and (A–C) Ch4 volumes and (D–F) hippocampal volumes in older individuals with MCI and AD, stratified by Aβ status. The red dashed lines illustrate the cut‐off value for MTL+, estimated as 1.5 standard deviations above the mean of cognitively unimpaired Aβ− individuals. The magnitude and direction of associations are represented by Spearman correlation coefficient *ρ*. Aβ, amyloid beta; Aβ−, amyloid beta burden within normal limits; Aβ+, abnormal amyloid beta burden; Ch4, nucleus basalis of Meynert; CU, cognitively unimpaired; AD, Alzheimer's disease; MCI, mild cognitive impairment; MTL, mesial temporal region; MTL+, abnormal mesial temporal region CenTauR measure;

Among participants with MCI and AD, analysis revealed significant associations between MTL CenTauR values and Ch4 volumes in both MCI Aβ− (*n* = 124; *ρ* = −0.280, *P* = 0.002; Figure 4A) and MCI Aβ+ groups (*n* = 259; *ρ* = −0.344, *P* < 0.001; Figure 4B), but not in the AD group (*n* = 61; *ρ* = −0.182, *P* = 0.160; Figure 4C). A similar pattern was observed for hippocampal volumes, with associations of equivalent magnitude across all three symptomatic subgroups (Figure 4D–F). These observed between‐group differences in the associations between MTL CenTauR values and brain volume, spanning CU Aβ−, CU Aβ+, MCI Aβ+, and AD groups, were further confirmed by the moderation analyses (Table S1 in supporting information).

The contribution of the covariance of Aβ and tau PET quantifications to the observed associations in the MCI Aβ+ group was re‐evaluated, showing that the covariance of Aβ CL and MTL CenTauR values partially accounted for the associations between MTL CenTauR and Ch4 volumes (β[SE] = −0.069[0.033], *P* = 0.037; Figure 3B) as well as hippocampal volumes (β[SE] = −0.090[0.033], *P* = 0.006; Figure 3C). Additionally, MTL CenTauR values exhibited a significant direct effect on Ch4 and hippocampal volumes, independent of Aβ CL values (both *P* < 0.001).

### 3.5 Association of TE tau burden and brain volume in symptomatic AD

As tau pathology typically spreads to the neocortex with the emergence of cognitive symptoms,^34^ we further examined the associations between TE tau burden and brain volumes in cognitively impaired individuals. Significant associations between TE CenTauR and Ch4 and hippocampal volumes were observed only in the MCI Aβ+ group, whereas no significant relationships were found in the MCI Aβ− or AD groups (Figure S2 in supporting information).

Moreover, correlation analyses were also repeated using Ch4p volume, which yielded results largely consistent with those based on Ch4 volume, with minor differences observed for the association with Aβ burden in the CU MTL+ group and with MTL tau burden in the AD group (Table S2 in supporting information).

### 3.6 Voxel‐wise associations between tau PET and brain volumes

**FIGURE 5:**
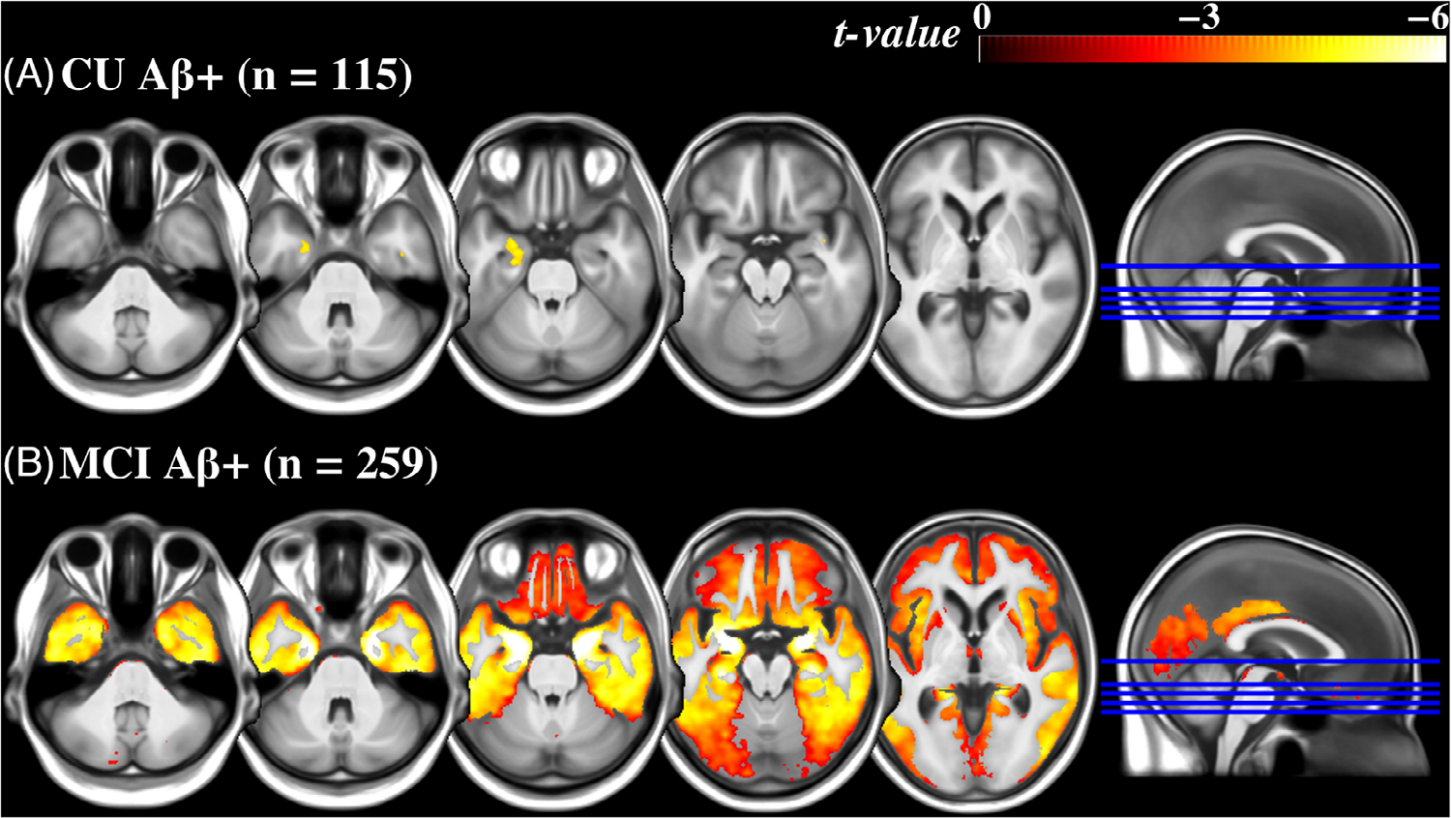
Voxel‐wise associations (FDR corrected *P* < 0.05) between tau PET SUVR and Ch4 volume observed in the (A) CU Aβ+ and (B) MCI Aβ+ groups. Voxel‐wise general linear models were performed with ^18^F‐MK6240 SUVR maps as the dependent variable and Ch4 volume as the independent variable. Ch4 volume was pre‐adjusted for total intracranial volume, age, and sex, and no additional covariates were included. The colormap displays the *t* values of the observed voxel‐wise associations, ranging from 0 to −6, indicating the statistical significance of the predictor's effect. Aβ+, abnormal amyloid beta burden; Ch4, nucleus basalis of Meynert; CU, cognitively unimpaired; FDR, false discovery rate; MCI, mild cognitive impairment; PET, positron emission tomography; SUVR, standardized uptake value ratio

Voxel‐wise analyses revealed that within the CU Aβ+ group, smaller Ch4 volume was associated with higher tau PET SUVR primarily in the left amygdala and hippocampus (*P* < 0.05, FDR corrected; Figure 5A). A similar pattern was observed when including the entire CU group (Figure S3A in supporting information). In the MCI Aβ+ group, the associations were much more widespread, extending beyond the temporal cortex into parietal and fontal cortices (Figure 5B). In the AD group, no significant negative associations survived correction. When the analyses were repeated using hippocampal volume, similar patterns were observed only in the MCI Aβ+ group (Figure S3B), whereas the CU Aβ+ and AD groups showed no significant associations after correction.

## 4 DISCUSSION

This cross‐sectional study presents several lines of evidence suggesting that MTL tau pathology plays an important role in BF cholinergic degeneration in preclinical and prodromal stages of AD: (1) The effect of Aβ burden on Ch4 volume was significant in CU MTL+ individuals but not in CU MTL− individuals, highlighting the concurrence of Aβ and MTL tau pathology in association with early Ch4 volume loss. (2) No association was found between MTL CenTauR values and Ch4 volumes in the CU group; rather, this relationship emerged exclusively in CU Aβ+ individuals, suggesting that Aβ pathology may be necessary for the tau–Ch4 association. (3) The association between MTL tau burden and Ch4 volumes was strongest in MCI Aβ+ individuals (*ρ* = −0.338). Importantly, this effect of MTL CenTauR on Ch4 volume remained significant after accounting for the indirect effect of Aβ CL values.

Previous studies have consistently demonstrated an association between Aβ burden and BF atrophy in CU older individuals, highlighting the early and selective vulnerability of the cholinergic system.^14^, ^15^, ^17^, ^18^ We replicated this finding in our CU cohort, showing a significant negative association between Aβ CL values and Ch4 volumes. Additionally, in preclinical AD, higher MTL CenTauR values were associated with smaller Ch4 volumes. Notably, no significant associations were found between hippocampal volumes and either Aβ or tau burden in CU individuals. These results provide further evidence that Ch4 degeneration occurs early, preceding hippocampal atrophy, a characteristic marker of neurodegeneration in AD, and highlight the link between Ch4 vulnerability and MTL tau pathology in the preclinical phase of the disease.

The role of tau pathology in early AD‐related BF degeneration has been largely overlooked in in vivo imaging studies, likely due to the limited availability of reliable tau measures. Our ^18^F‐MK6240 PET data revealed that ≈ 25% of CU individuals had MTL CenTauR measures exceeding the cut‐off, defined as 1.5 SD above the mean of CU Aβ− individuals. The association between Aβ burden and Ch4 volumes was slightly attenuated in CU TE− individuals and was not significant in CU MTL− individuals. These findings indicate that the Aβ–Ch4 relationship is modulated by the presence of MTL tau pathology and may be further strengthened by increased pathological tau burden including early neocortical tau. Additionally, mediation analysis indicates that Ch4 degeneration, assessed by MRI‐based volumetric measures, is closely linked to the co‐occurrence of Aβ and MTL tau pathology, with the covariance of their PET measures accounting for 57.5% of the total effect of Aβ burden on Ch4 volume.

When directly examining the association between MTL CenTauR and Ch4 volumes, we found no significant association in the entire CU group. However, in CU Aβ+ individuals, higher MTL CenTauR values were associated with smaller Ch4 volumes, suggesting that the presence of established Aβ pathology may be necessary for the link between early tau pathology and Ch4 degeneration. Notably, this association was not significantly explained by the indirect effect through Aβ CL values. These data suggest that Ch4 volume appears more closely associated with MTL tau quantification than with Aβ burden, potentially indicating a more direct biological link between tau pathology and BF degeneration. However, this finding may also result from differences in the goodness of fit of imaging measures, which cannot be confirmed in vivo.

On the other hand, no significant association between MTL CenTauR and Ch4 volumes was found in CU Aβ− individuals. However, with the emergence of MCI (CDR = 0.5), higher MTL CenTauR values were associated with smaller volumes of Ch4 and hippocampus in Aβ− individuals. The results indicate that in the absence of established Aβ pathology, MTL tau pathology does not contribute to Ch4 degeneration in aging CU individuals. Instead, Ch4 atrophy may be a downstream consequence, emerging alongside hippocampal atrophy and cognitive symptoms, or reflect the prodromal stage of a different dementia type. An alternative explanation for the lack of association in CU Aβ− individuals may be due to their limited range of MTL CenTauR and a low prevalence of CU individuals with primary age‐related tauopathy,^38^ which may preclude the detection of any relationship.

The association between MTL CenTauR measures and Ch4 volumes emerges early in preclinical AD (*ρ* = −0.263), and peaks in the prodromal stage among MCI Aβ+ individuals (*ρ* = −0.338). However, as the disease progresses to the dementia stage, the association becomes less pronounced (*ρ* = −0.181), possibly indicating a plateau effect, in which Ch4 volume loss might have already largely occurred. These findings were further supported by voxel‐wise analyses (Figure 5), which showed that the associations emerged in the amygdala and hippocampus in preclinical AD and became widespread during the prodromal stage. A similar pattern was observed for tau burden in the TE, with a strong association between TE CenTauR and Ch4 volumes in the MCI Aβ+ group, which became notably attenuated in the AD group. This pattern suggests that the preclinical and prodromal stages may represent a critical window for early intervention aimed at enhancing the cholinergic function and/or preserving the integrity of the BF cholinergic system.

Our findings should be interpreted with caution, due to some limitations. Ch4 volumetric measures capture gross tissue changes that are relative to cholinergic neuronal loss. Our findings remain inconclusive regarding the extent of BF cholinergic neuron loss that may be attributed to Aβ pathology alone before MTL tau emerges. Moreover, global scaled measures such as CL values may not fully capture regionally specific cortical or subcortical pathology relevant to these processes. Future work incorporating high‐resolution PET imaging of regional Aβ and tau pathology, particularly within the BF^39^ and hippocampus, together with cholinergic PET tracers,^40^, ^41^ will be essential to clarify these early proteinopathy‐related effects on the cholinergic system. The use of predefined cut‐offs for determining Aβ− or MTL− individuals may not entirely exclude the subthreshold pathology. Nonetheless, no significant associations were observed in CU Aβ− or CU MTL− individuals, indicating minimal impact. AIBL participants represented a well‐educated volunteer cohort with few comorbidities due to exclusion criteria, who were not randomly selected from the community, potentially limiting generalizability beyond similar populations.

In conclusion, our study provides evidence that the co‐occurrence of Aβ and MTL tau pathologies significantly contributes to reduced Ch4 volume in the preclinical stage of AD. We highlight a strong association between Ch4 degeneration and early tau pathology in the MTL exclusively in CU individuals with established Aβ pathology, which was not observed for the hippocampus. This relationship persists into the prodromal stage of AD, as tau pathology spreads into the neocortical regions. These findings underscore the potential cognitive benefits of cholinergic therapies in early disease stages.

## CONFLICT OF INTEREST STATEMENT

All authors report no conflicts of interest relevant to this article. Author disclosures are available in the supporting information.

## CONSENT STATEMENT

The AIBL study was approved by institutional ethics committees of Austin Health, St. Vincent's Health, Hollywood Private Hospital, and Edith Cowan University. Written informed consent was obtained from all volunteers before participation and at each visit.

## Supporting information



Supporting information

Supporting information
