# Supplementary material for: Mesial temporal tau pathology impacts basal forebrain degeneration in early Alzheimer's disease
Source: Alzheimers Dement. 2025 Dec 26;21(12):e71050. doi: 10.1002/alz.71050 (PMC12741919; doi:10.1002/alz.71050)
Supplement: Supplementary file 2 — Supporting information [file ALZ-21-e71050-s002.pdf]

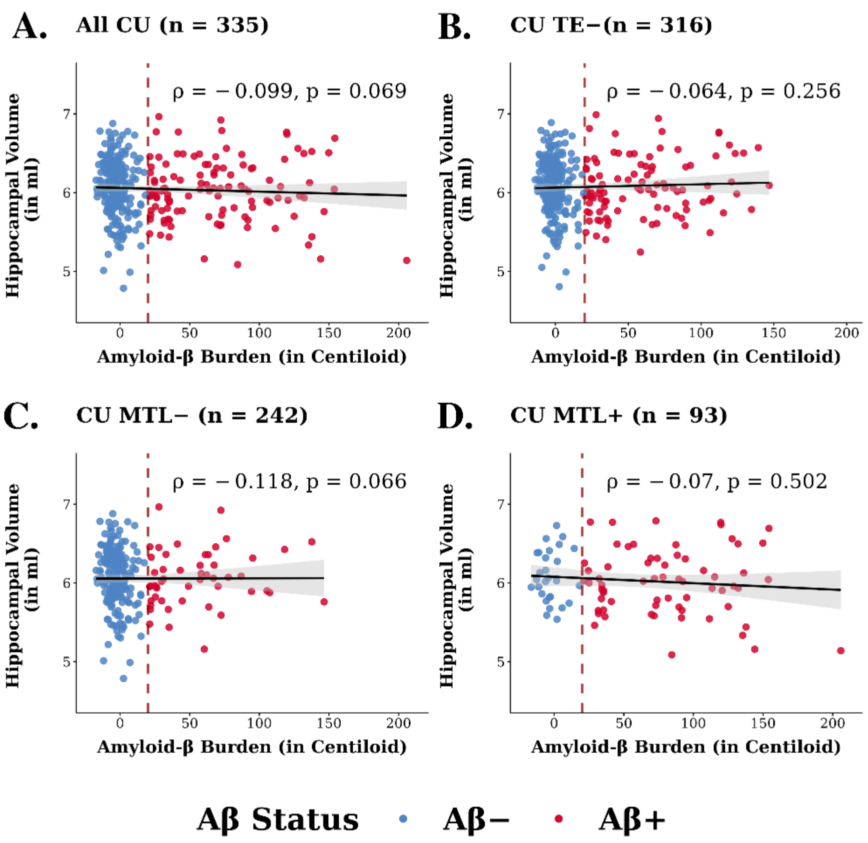

eFigure 1: Associations between Aβ burden (in Centiloid) and hippocampal volumes in (A) CU older individuals, (B) CU TE− individuals, and (C-D) subgroups stratified by MTL tau status. The red dashed lines illustrate the cut-off value of 20 Centiloids applied for Aβ+. The magnitude and direction of associations are represented by Spearman's correlation coefficient ρ. Abbreviations: Aβ, amyloid-β; Aβ+, abnormal Aβ burden; CU, cognitively unimpaired; MTL, the mesial temporal region; MTL−, MTL CenTauR within normal limits; MTL+, abnormal MTL CenTauR measure; TE, the temporo-parietal region; TE−, TE CenTauR within normal limits.

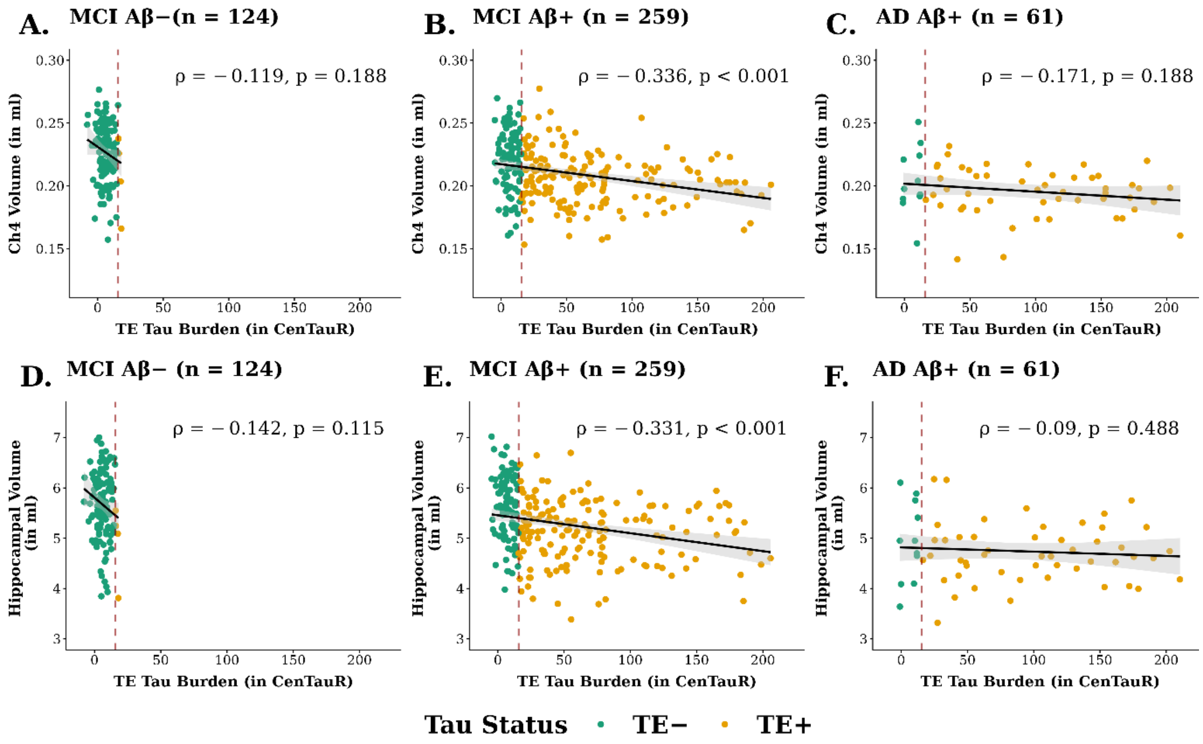

eFigure 2: Associations between TE CenTauR measures and (A-C) Ch4 volumes and (D-F) hippocampal volumes in older individuals with MCI and AD, stratified by Aβ status. The red dashed lines illustrate the cut-off value for TE+, estimated as 2.0 standard deviations above the mean of cognitively unimpaired Aβ− individuals. The magnitude and direction of associations are represented by Spearman's correlation coefficient ρ. Abbreviations: Aβ, amyloid-β; Aβ−, Aβ burden within normal limits; Aβ+, abnormal levels of Aβ burden; AD, Alzheimer's disease; MCI, mild cognitive impairment; Ch4, nucleus basalis of Meynert; TE, the temporo-parietal region; TE+, abnormal TE CenTauR measure.

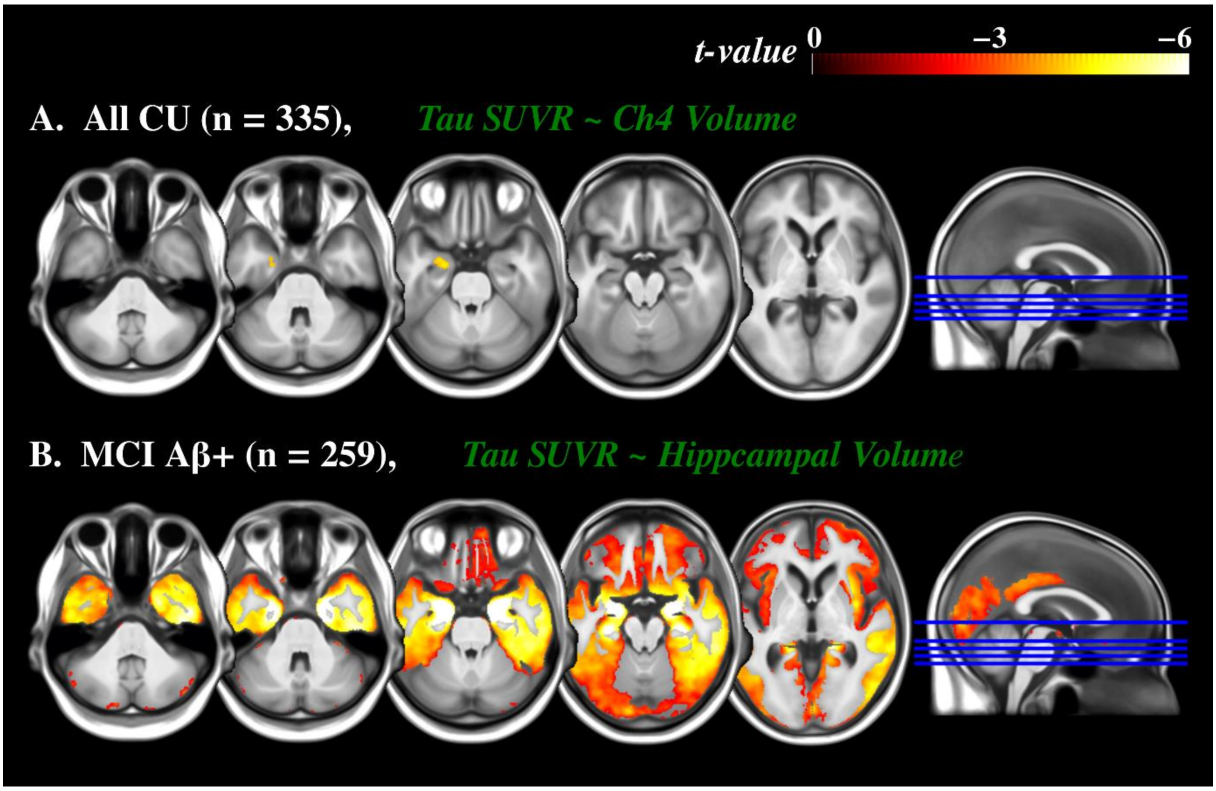

eFigure 3: Voxel-wise analysis (FDR-corrected $p < 0.05$) showing (A) associations between tau PET SUVR and Ch4 volume in all CU individuals and (B) associations between tau PET SUVR and hippocampal volume in the MCI Aβ+ group. Voxel-wise general linear models were performed with ^{18}F-MK6240 SUVR maps as the dependent variable and Ch4 or hippocampal volume as the independent variable. The colormap displays the *t*-values of the observed voxel-wise associations, ranging from 0 to −6, indicating the statistical significance of the predictor's effect. Abbreviations: Aβ+, abnormal Aβ burden; Ch4, nucleus basalis of Meynert; CU, cognitively unimpaired; FDR, false discovery rate; MCI, mild cognitive impairment; PET, positron emission tomography; SUVR, standardized uptake value ratio.

eTable 1. Moderation analyses testing group effects on the association between MTL tau burden and Ch4 or hippocampal volume.

**Region**	**Term**	**Estimate**	**Std. Error**	***p* value**
Reference Group: CU Aβ−				
**Ch4**	Ch4	0.031	0.131	0.811
	Ch4 × Group (CU Aβ+)	−0.396	0.215	0.066
	Ch4 × Group (MCI Aβ+)	−0.644	0.153	< 0.001
	Ch4 × Group (AD Aβ+)	−0.486	0.227	0.033
**Hippocampus (HV)**	HV	−0.022	5.528	0.997
	HV × Group (CU Aβ+)	−3.780	8.976	0.674
	HV × Group (MCI Aβ+)	−23.281	6.198	< 0.001
	HV × Group (AD Aβ+)	−14.646	8.231	0.076

Models were fit using general linear modeling with MTL tau burden (CenTauR values) as the dependent variable. Formula: MTL tau ~ Brain Volume + Group + Brain Volume : Group, where Group is a categorical factor (CU Aβ−, CU Aβ+, MCI Aβ+, AD Aβ+). Ch4 and hippocampal volumes were pre-adjusted for total intracranial volume, age, and sex.

Abbreviations: Aβ, amyloid-β; Aβ−, Aβ burden within normal limits; Aβ+, abnormal levels of Aβ burden; AD, Alzheimer’s disease; CU, cognitively unimpaired; MCI, mild cognitive impairment; Ch4, nucleus basalis of Meynert; MTL, the mesial temporal region.

eTable 2. Correlation analyses repeated using Ch4p volume to examine potential subregional effects within the basal forebrain.

	No.	vs. Aβ burden (in Centiloid)	
**All CU**	n = 335	ρ = −0.127, p = 0.020	
**CU TE−**	n = 315	ρ = −0.087, p = 0.123	
**CU MTL−**	n = 242	ρ = −0.082, p = 0.206	
**CU MTL+**	n = 93	ρ = −0.166, p = 0.111	
		**vs. MTL tau (in CenTauR)**	**vs. TE tau (in CenTauR)**
**All CU**	n = 335	ρ = −0.021, p = 0.695	-
**CU Aβ−**	n = 220	ρ = 0.118, p = 0.082	-
**CU Aβ+**	n = 115	ρ = −0.210, p = 0.025	-
**MCI Aβ−**	n = 124	ρ = −0.241, p = 0.007	ρ = −0.114, p = 0.209
**MCI Aβ+**	n = 259	ρ = −0.366, p < 0.001	ρ = −0.332, p < 0.001
**AD Aβ+**	n = 61	ρ = −0.262, p = 0.042	ρ = −0.170, p = 0.189

Spearman's rho (ρ) and p-values were reported. Abbreviations: Aβ, amyloid-β; Aβ−, Aβ burden within normal limits; Aβ+, abnormal levels of Aβ burden; AD, Alzheimer's disease; CU, cognitively unimpaired; MCI, mild cognitive impairment; Ch4p, posterior subdivision of nucleus basalis of Meynert; MTL, the mesial temporal region; MTL−, MTL CenTauR within normal limits; MTL+, abnormal MTL CenTauR measure; TE, the temporo-parietal region; TE−, TE CenTauR within normal limits.
